# Deep reinforcement learning with significant multiplications inference

**DOI:** 10.1038/s41598-023-47245-y

**Published:** 2023-11-27

**Authors:** Dmitry A. Ivanov, Denis A. Larionov, Mikhail V. Kiselev, Dmitry V. Dylov

**Affiliations:** 1 Lomonosov Moscow State University, GSP-1, Leninskie Gory, Moscow, 119991 Russia; 2 Cifrum, 3 Kholodil’nyy per., Moscow, 115191 Russia; 3 Chuvash State University, 15 Moskovsky pr., Cheboksary, Chuvash Republic 428015 Russia; 4 Skolkovo Institute of Science and Technology, 30/1 Bolshoi blvd., Moscow, 121205 Russia; 5 Artificial Intelligence Research Institute, 32/1 Kutuzovsky pr., Moscow, 121170 Russia

**Keywords:** Computational science, Computer science

## Abstract

We propose a sparse computation method for optimizing the inference of neural networks in reinforcement learning (RL) tasks. Motivated by the processing abilities of the brain, this method combines simple neural network pruning with a delta-network algorithm to account for the input data correlations. The former mimics neuroplasticity by eliminating inefficient connections; the latter makes it possible to update neuron states only when their changes exceed a certain threshold. This combination significantly reduces the number of multiplications during the neural network inference for fast neuromorphic computing. We tested the approach in popular deep RL tasks, yielding up to a 100-fold reduction in the number of required multiplications without substantial performance loss (sometimes, the performance even improved).

## Introduction

Modern deep learning (DL) gravitates towards large neural networks, with ever-increasing demands for computational resources to perform basic arithmetic operations, such as multiplication. When used with contemporary DL hardware, known to face the limitation of the von Neumann bottleneck^1^, this results in a substantial energy consumption and significant delays during the network inference. At the same time, the human brain is capable of inferring with remarkable efficiency by dismissing the irrelevant signals and connections, consuming just 10–20 W for basic cognitive tasks^2^. Such efficiency motivated our study to optimize the DL inference by taking into account *only significant* signals. Specifically, we consider the inference in RL tasks, using the popular Atari games environment^3^ as the sandbox.

In the brain, the presence of regular dense layers is not evident. Instead, the brain employs neural rewiring as a means to eliminate inefficient and unnecessary connections^4^. At the same time, recent studies^5–7^ demonstrate that in many cases significant part of neural connections are excessive and can be removed without a drop in the neural network’s predictive power (or with a negligible one). The optimization technique, known as pruning, represents a method to attain structural sparsity within a neural network. The conceptualization of this approach can be traced back to the 1990s^8,9^. Today, there are multiple strategies for identifying redundant connections within neural networks, including the examination of absolute values, the Hessian matrix, decomposition methods, and others^5,10^.

Moreover, neural networks often anticipate the input data in a form of sequential highly correlated signals or frames, as observed in domains like video, audio, monitoring and control problems, including RL. In such tasks, the information processed by a neural network at time step *t* closely resembles what it analyzed at the time step $t-1$. This phenomenon is referred to as temporal sparsity, also characteristic of the perceptual systems in the brain^4,11^. Some studies propose various optimization algorithms for neural networks handling such temporal sparse data^12–15^. These approaches are based on the idea of asynchronous updates, where only the states of neurons that have changed significantly, compared to the previous step, are updated. It is worth noting that the brain neurons also operate asynchronously, transmitting signals to each other only when necessary.

A typical neural network layer could be represented as a matrix-vector multiplication combined with the application of a nonlinear transformation. Therefore, for the input vector *x*, the $j^{\text {th}}$ value of the output vector *y* could be represented as

$$\begin{aligned} y^j = f(w_1^j * x_1 + \dots + w_n^j * x_n), \end{aligned}$$

where *f* is a nonlinear function applied to the Multiply and Accumulate (MAC) operation. The MAC operation is the summation of products of the input vector elements and the respective weights *w*. If at least one operand in any of such multiplications is zero, then such multiplication could be omitted. In this work, we refer to a multiplication of two numbers as *significant* if neither operand is equal to zero.

In this study, we propose a combination of the two aforementioned approaches to optimize the inference of Neural Networks in RL tasks with the video inputs. More specifically, we optimize the inference of a popular DQN algorithm^16^ for the Atari games. The pruning approach yields a 2–8$\times$ reduction in the number of significant neural network multiplications. The second part of our approach achieves a further 10–50-fold reduction. When combined, these approaches result in a 20–100-fold reduction in the number of significant multiplications, without notable performance loss and in some cases, even enhancing the original performance.

The proposed combination of these methods is biologically inspired and exhibits neuromorphic properties for fast information processing^1^. To the best of our knowledge, such an approach has never been applied in deep RL problems.

## Background

### Deep Q-network

In RL tasks^17^, an agent receives the current environment state *s* as an input, after which it selects action *a*, and then it goes to a new state $s'$, and it receives a certain reward *r*. The agent’s goal is to maximize the total reward.

More strictly, the environment is formalized as a Markov decision process. A Markov decision process (MDP) is a tuple (*S*, *A*, *P*, *R*), where *S* is a set of possible states and *A* is a set of possible actions. *P* is the function describing transition between states; $P_{a}(s, s') = Pr(s_{t+1} = s' | s_t = s, a_t = a)$, i.e., the probability to get into state $s'$ at the next step when selecting action *a* in state *s*. $R = R_{a}(s,s')$ is the function describing receiving rewards. It determines how big a reward an agent will receive when transitioning from state *s* to state $s'$ by selecting action *a*.

The strategy an agent uses to select its actions *a* depending on state *s* is called a policy and is usually denoted by the letter $\pi _\theta$ where $\theta$ denotes policy parameters.

One way to solve an RL task is *Q*-function-based approaches. A *Q*-function has two parameters - *s* and *a*. $Q_{\pi _\theta }(s,a)$ estimates what cumulative reward the agent following policy ${\pi _\theta }$ will receive if it performs action *a* from state *s* and then follows policy ${\pi _\theta }$. If complete information about an environment is available, the exact value of a *Q*-function can be calculated. However, the knowledge of the world is usually incomplete and the number of possible states is enormous. Therefore, the *Q*-function can be approximated using neural networks. This approach named DQN (Deep Q-Network) was demonstrated by DeepMind in^16^. In the present study, we use this algorithm to train Neural Networks for Atari RL tasks.

### Pruning and lottery ticket hypothesis

The authors of^7^ have proposed the Lottery Ticket Hypothesis. The hypothesis states that when at any stage of neural network training the smallest weights by absolute value are pruned (set to zero and frozen) and the remaining weights are reset to the values they had before the training, and then neural network training is resumed, the training capabilities of such neural network will remain the same. However, this does not happen if we set the remaining weights to random (not initial) values. Furthermore, the investigation reveals that sparse neural networks, which have undergone such pruning and training, can exhibit superior performance compared to unpruned neural networks.

**Figure 1:**
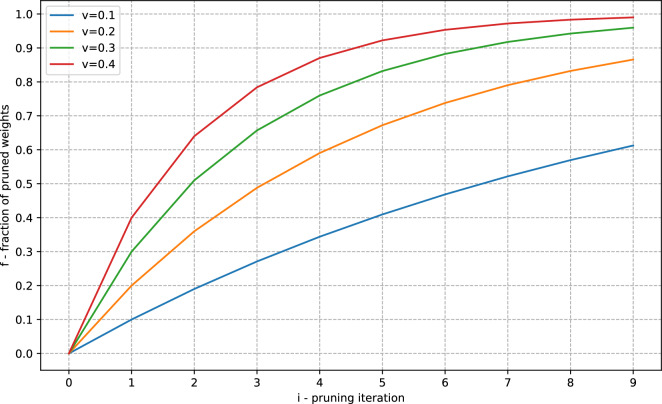
Dependencies of the fraction of pruned weights on pruning iterations *i* for various pruning rates *v*.

In practice^7,18,19^ neural network iterative magnitude pruning by absolute value is usually used. This approach involves training neural networks, followed by pruning a small percentage of their weights (typically 10-20%). The remaining weights are then reset to their initial values, and this process is repeated for a specified number of iterations, denoted as *M*. Consequently, given a pruning rate of *v*, the fraction *f* of pruned weights can be computed as follows:

1 $$\begin{aligned} f = \frac{pruned\_weights}{total\_weights} = 1 - (1-v)^i,\quad i \in [0:M) \end{aligned}$$

where *i* is the pruning iteration. Figure 1 displays the relationships between the fraction of pruned weights and various values of *i* and *v*.

In^18,19^ it was shown that the phenomenon of the Lottery Ticket Hypothesis is observed in RL tasks as well. In particular, the authors of^19^ investigated this phenomenon within the context of the DQN algorithm.

### DeltaNetwork algorithm

The output values of the neural network layer number $k+1$ can be written as:

2 $$\begin{aligned} o^{k+1}= & {} W^{k} x^{k} + b^{k} \end{aligned}$$

3 $$\begin{aligned} x^{k+1}= & {} f(o^{k+1}) \end{aligned}$$

where $x^{k+1} \in R^n$ is the output of $k+1$ neural network layer, $x^k \in R^n$ is the input of $k+1$ neural network layer (output of *k* layer), $W^{k} \in R^{nxn}$ is the weight matrix, and $b^{k} \in R^{n}$ is the bias. In a conventional neural network, for every new input vector $x^{k}(t)$ in the moment of time t a total recomputation of output value $x^{k+1}(t)$ is required, which will require $n^2$ multiplications. However, the following should be noted:

4 $$\begin{aligned} \Delta x^k(t)= & {} x^k(t) - x^k(t-1) \end{aligned}$$

5 $$\begin{aligned} o^{k+1}(t)= & {} W^{k} \Delta x^{k}(t) + o^{k+1}(t-1) \end{aligned}$$

6 $$\begin{aligned} \Delta x^{k+1}(t)= & {} f(o^{k+1}(t)) - f(o^{k+1}(t-1)) \end{aligned}$$

Thus, it is possible to recompute layer output values at the moment of time t using Eqs. (4), (5), (6) using layer input changes that occur relative to the state at the moment $t-1$.

This remark does not lead to neural network optimization by itself. But we can introduce threshold *T* for output value changes $\Delta x^{k}(t)$ such that recomputation of succeeding neurons is started only when an output value exceeds this threshold.

Authors of^12,13^ call this approach “Hysteresis Quantizer”. To implement it, it is necessary to introduce an additional variable $x\_prev(t)$ into each neuron. Such a variable will be used to record the last transmitted value. Thus, the following Algorithm 1 will be run on each neuron:

**Algorithm 1:**
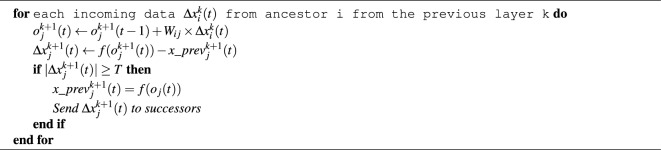
Update neuron j of layer k+1 at time t.

### Related works

To the best of our knowledge, we present the first attempt to combine the temporal and the structural sparsity for optimizing inference in RL tasks. However, there are several publications that applied pruning^18,20^ and other optimization techniques to RL problems, e.g., distillation^21^. Also, there are works that employ the DeltaNetwork algorithm for convolutional and recurrent neural networks^13,22,23^.

**Figure 2:**
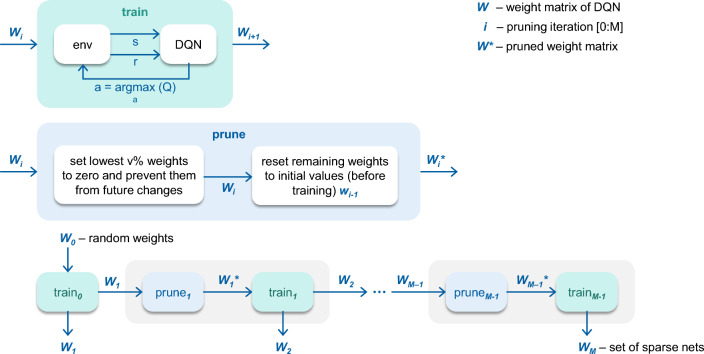
The pipeline, including the training and the pruning stages.

## Methods

In the previous section, we described two neural network optimization algorithms: Pruning and DeltaNetwork Algorithm. The following neural network optimization becomes possible when these approaches are applied jointly:

**Stage 1—Training:**

1. Train neural network in the environment
2. Prune (set to zero and freeze) lowest $v = 20$% weights
3. Reset remaining weights to original
4. Train neural network again in the environment
5. Repeat steps 2–4 $M = 10$ times

Using this algorithm, we obtain a set of structurally sparse neural networks with different degrees of sparsity. The number of neural networks in the set equals the number of pruning algorithm iterations. The general scheme of the learning stage of the algorithm is shown in Fig. 2.

**Figure 3:**
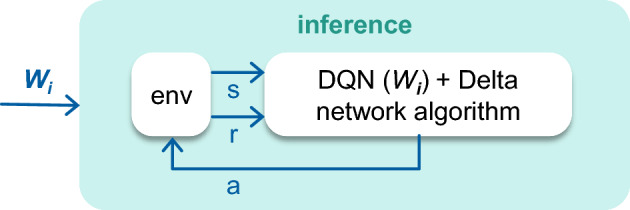
The inference stage of the algorithm.

**Stage 2—Inference**: Then we apply the DeltaNetwork Algorithm with the threshold $T = 0.01$ to the inference of the trained neural networks. As a result, we get a set of new neural networks using both structure and temporal sparsity. The selection of one neural network from such a set depends on the desirable balance between the number of significant multiplications and neural network performance. The scheme of the inference stage is presented in Fig. 3.

## Experiments

### Neural network architecture

In this study, we conducted all experiments using the following Neural Network architecture.

**Figure 4:**
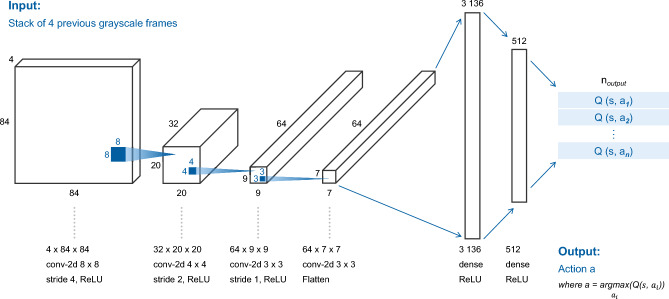
Optimized DQN architecture. DQN consists of three convolutional layers and two dense layers. This architecture is suitable for all video games (the number of outputs at the last layer is the only value that changes).

An 84 $\times$ 84 $\times$ 4 matrix received from the environment is an input to the neural network, which consists of 4 sequential game frames. The first convolutional layer consists of 32 8 $\times$ 8 filters with strides equal to 4 and ReLU activations. The second layer consists of 32 4 $\times$ 4 filters with strides equal to 2 and ReLU activations. The third layer consists of 64 3 $\times$ 3 filters with strides equal to 2 and ReLU activations. They are followed by a dense layer with 512 neurons with ReLU activations. At the output there is another dense layer with a number of neurons equal to the number of actions in a video game. Depending on the video game, the number of actions *n* may vary from 4 to 18. The neural network structure is shown in Fig. 4.

### RL environments

**Figure 5:**
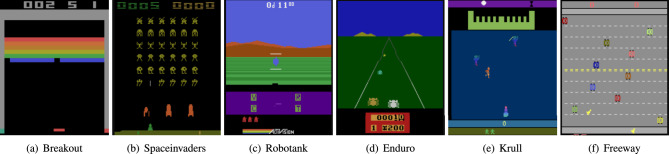
RL environments for experiments.

We experimented within the following RL environments: Breakout, SpaceInvaders, Robotank, Enduro, Krull and Freeway. Figure 5 shows frames from these video games. For example, in Breakout, an agent has to hit as many bricks as possible by hitting the ball. In Spacelnvaders, an agent has to eliminate all alien spaceships.

### Significant operations counting

#### The number of multiplications before optimization

Let us estimate the number of multiplications in a standard neural network without any optimizations. The following formula can be used to estimate the number of multiplications in a convolutional layer *k*:

7 $$\begin{aligned} {\gamma _k} = size\_x_{k+1} * size\_y_{k+1} * {filters_{k+1}}* kernel\_size\_x_{k} * kernel\_size\_y_{k} * filters_{k}, \end{aligned}$$

where

- $size\_x_{k+1}$—is the $k+1$ layer input size along the *x* axis
- $size\_y_{k+1}$—is the $k+1$ layer input size along the *y* axis
- $filters_{k+1}$—is the number of filters at layer *k* ($k+1$ layer input size along the *z* axis)
- $kernel\_size\_x_{k}$—is the convolution size along the *x* axis
- $kernel\_size\_y_{k}$—is the convolution size along the *y* axis
- $filters_{k}$—is the *k* layer input size along the *z* axis

The following formula can be used to count multiplications in dense layer *k*:

8 $$\begin{aligned} {\gamma _k} = input_{k} * output_{k}, \end{aligned}$$

where

- $input_{k}$—is the *k* layer input size (number of neurons at $k-1$ layer)
- $output_{k}$—is the *k* layer output size (number of neurons at *k* layer)

General results for all layers are presented in Table 1. It should be noted that these results are universal for any Atari environment and for any DQN run.

**Table 1:** The structure of the DQN network and the number of multiplications in each layer before optimization.

Layer	Input shape	#Param	#Multiplications
Conv2d-1 (8 $\times$ 8, stride = 4)	[4, 84, 84]	8224	3,276,800
Conv2d-2 (4 $\times$ 4, stride = 2)	[32, 20, 20]	32,832	2,654,208
Conv2d-4 (3 $\times$ 3, stride = 1)	[64, 9, 9]	36,928	1,806,336
Flatten	[64, 7, 7]	0	0
Dense-1 (3136, 512)	[3136]	1,606,144	1,605,632
Dense-2 (512, $n_{output}$)	[512]	$512 * n_{output} + n_{output}$	$512 * n_{output}$

#### The number of multiplications after optimization

It is clear that the degree of weight sparsity will affect the number of non-zero multiplications. However, the delta algorithm provides different levels of temporal sparsity depending on the selected threshold, layer, and input data. That is why it is impossible to analytically estimate the number of non-zero multiplications. Therefore we empirically calculated the number of significant multiplications by calculating all operations that have zero operands. Examples of the numbers of significant multiplications are given in the next section and in Tables 2 and 3.

## Results

**Figure 6:**
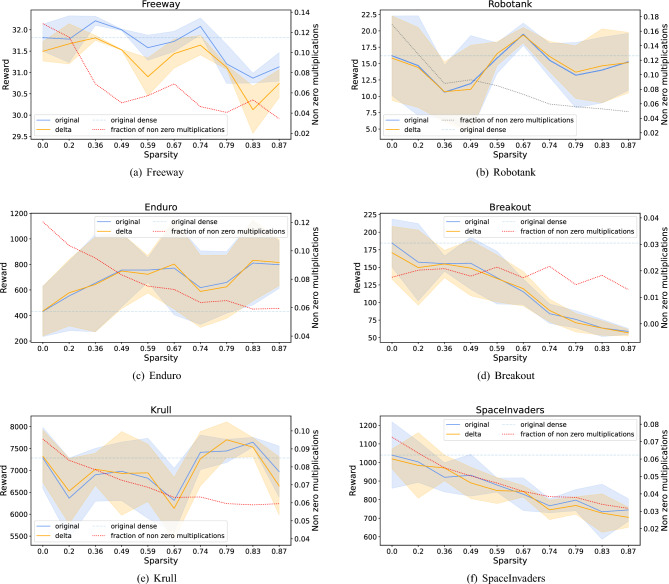
Results for Freeway, Robotank, Enduro, Breakout, Krull and SpaceInvaders. The *x* axes of the figures denote the neural network sparsity degree; the left *y* axes denote the reward received by an agent; the right *y* axes denote fraction of significant multiplications averaged by environment runs. The orange line shows the performance of the pruned network, while the blue line shows the performance of the pruned network with the additional application of the DeltaNetwork algorithm. The grey dotted lines show the fraction of significant multiplications (the less the better) of a pruned neural network enhanced by the DeltaNetwork algorithm. The blue dashed lines demonstrate the performance of a neural network without any optimization.

Figure 6 shows the abovementioned neural network performance metrics at different sparsity levels and for different environments.

The reward metric results (blue line) are similar to the results from^18,19^, where the highest decrease of reward during pruning was in SpaceInvaders and Breakout. In these environments performance dropped very quickly as neural network sparsity grew. At the same time for Enduro the reward during the pruning does not decrease. It even increases. For Robotank and Freeway the performance does not degrade seriously, and even for some sparsity levels it increases in comparison with the unpruned version. Thus it is possible to use very sparse neural networks for some environments without the loss of performance.

The orange line demonstrates the rewards for the neural networks with delta neurons. We can see that they have similar (sometimes a little bit higher, sometimes a little bit lower) performance to a network without delta neurons. This means that the delta algorithm does not influence the reward gravely.

The gain in the number of non-zero multiplications (black dotted line) is dependent on the game the agent is playing. For example, in SpaceInvaders the fraction of the non-zero multiplications is in the range from 0.065 to 0.022. At the same time in Breakout this value is in the range from 0.018 to 0.009.

The delta algorithm alone (without the weight pruning) leads to a decreasing in the number of non-zero multiplication operations from 5.5 times for Robotank to 55 times for Breakout. At the same time, the pruning leads to a more modest gain. For some environments, the fraction of non-zero multiplication operations decreases as a result of pruning more than for others. E.g., for Robotank the fraction of non-zero multiplication operations decrease during pruning from 0.184 to 0.059 (about x3.11). At the same time for Breakout it decreases only from 0.018 to 0.015 (x1.2). Anyway, it is clear that the gain of the delta algorithm is much higher than the gain of the pruning. Moreover, pruning sometimes leads to the degradation of performance.

We made 5 runs for every environment and averaged all metrics presented here. In the Supplementary Materials, the tables with the number of non-zero multiplication operations in layers are shown for each tested environment. In these tables, one can see that the highest gains occur in the first layers.

**Table 2:** Number of multiplications in one Breakout run with 0.79 sparsity and 0.01 threshold.

Layer	Multiplications	Non-zero multiplications	Fraction of zero multiplications	Sparsity of weights	Delta sparsity
Input	0	0	0.0	0.0	0.994
Conv2d-1	3,276,800	6554	0.998	0.600	0.974
Conv2d-2	2,654,208	13,271	0.995	0.778	0.927
Conv2d-4	1,806,144	19,868	0.989	0.843	0.949
Dense-1	1,605,632	80,281	0.950	0.0	0.815
Dense-2	2048	379	0.815	0.0	0.412
Total	9,344,832	121,483	0.987	0.79	0.977

**Table 3:** Number of multiplications in SpaceInvaders with 0.79 sparsity and 0.01 threshold.

Layer	Multiplications	Non-zero multiplications	Fraction of zero multiplications	Sparsity of weights	Delta sparsity
Input	0	0	0.0	0.0	0.986
Conv2d-1	3,276,800	16,384	0.995	0.693	0.935
Conv2d-2	2,654,208	45,122	0.983	0.764	0.802
Conv2d-4	1,806,336	70,447	0.961	0.835	0.857
Dense-1	1,605,632	228,000	0.858	0.0	0.756
Dense-2	2048	498	0.757	0.0	0.005
Total	9,344,832	364,448	0.961	0.79	0.943

## Discussion

**Figure 7:**
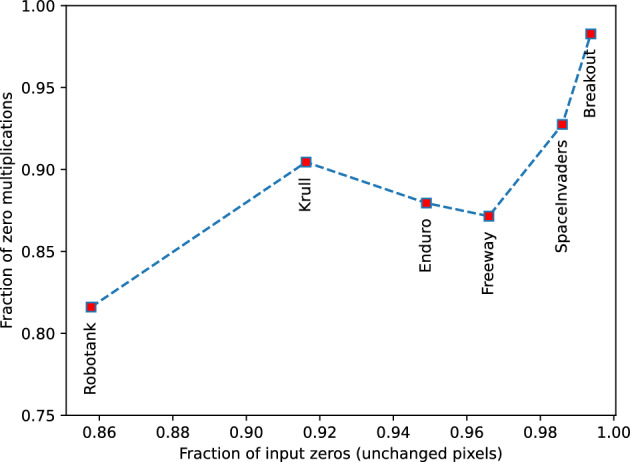
Correlation of the input sparsity (*x* axes) with zero multiplication fraction (*y* axes). Each red square corresponds to a particular environment.

The variety in performance gain between different environments can be explained by the fact that some environments (e.g., SpaceInvaders) have much more changing pixels at each time step than others (e.g., Breakout). In the Breakout only the playground and the ball move, while in the SpaceInvaders several objects can move at once—shots, a ship and aliens. This is well confirmed by the difference in delta sparsity (the fraction of neuron activations during the delta algorithm execution) level when playing Breakout and SpaceInvaders (see Tables 2 and 3). In Fig. 7, we visualized the correlation between the level of input sparsity caused by the delta algorithm with the fraction of zero multiplications. We see a tendency for the fraction of zero multiplications to increase during inference with an increase in the fraction of input zeros.

Both parts of the optimization approach (pruning and delta algorithm) presented here contribute to the reduction of the number of significant multiplications (see Fig. 6). Thus here we provided an efficient combination of structural and temporal sparsity.

**Figure 8:**
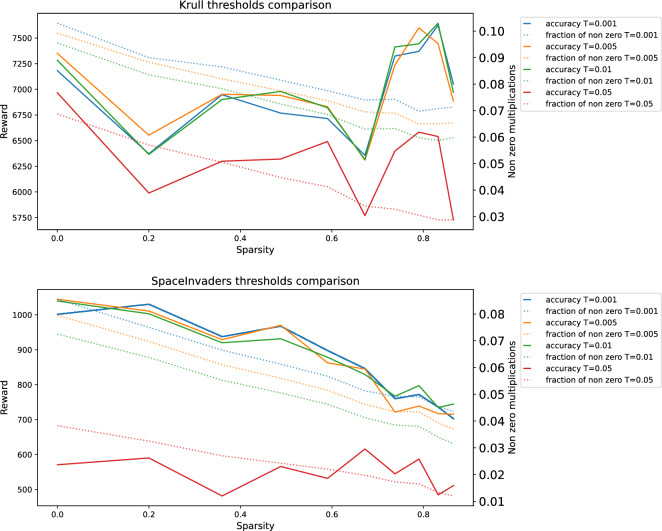
Comparison of thresholds for Krull and SpaceInvaders: The x-axis in the figures represents the degree of sparsity in a neural network. The left y-axes indicate the rewards achieved by the agent, while the right y-axes represent the fraction of significant multiplications. Dotted lines represent the percentage of significant multiplications (lower is better) in pruned neural networks enhanced by the DeltaNetwork algorithm. Solid lines depict the rewards of the agents. Different colors correspond to different thresholds.

Furthermore, we conducted an examination of how the parameter *T* (threshold) affects the performance and the operational gains. We assessed the performance in both Krull and SpaceInvaders using various values of *T*, namely 0.001, 0.005, 0.01, and 0.05. The results are presented in Fig. 8, clearly illustrating that 0.01 is the most favorable parameter value among those tested. The higher values, despite winning in the multiplication options, result in a significant decrease in the rewards. Whereas, the lower thresholds yield nearly identical rewards, with a more modest productivity improvement.

Multiplication is an expensive computational operation from the point of view of energy and time^24^. The desire to reduce the number of these operations is obvious. The provided algorithm decreases the number of significant multiplications. Moreover, due to the structural sparsity, this approach reduces the number of memory accesses, that are also very expensive in energy and time^24^.

However, presently, there are very few opportunities to use the existing hardware to effectively implement this algorithm. This is due to modern GPUs being designed for handling dense matrices. Nevertheless, there are attempts to turn the situation. Nvidia began offering hardware support of sparse matrix operations on one of its Tesla A100 GPUs; however, the maximum supported sparsity is only 75% so far^25^.

The authors of the abovementioned DeltaNetwork algorithm introduced the NeuronFlow^26,27^ architecture that supports delta algorithm. The Loihi2 processor that Intel presented^28^ in September 2021 also has the multi-core asynchronous architecture capable of running sparse delta neuron-based neural networks.

In^18^, it was shown that the Lottery Ticket Hypothesis pruning approach also works for RL algorithms trained by the A2C method. At the same time, DeltaNetwork Algorithm could be applied to any network working with sequential data. Thus, the RL algorithm for neural network training is not restricted by DQN.

In the future, it would be highly interesting to apply the proposed algorithm in new environments and real-world control tasks, assessing its performance on real hardware to measure the real efficiency gain. Additionally, it would be worthwhile to explore the potential enhancement of this algorithm through the incorporation of other neural network optimization methods, such as quantization, which can lead to reduced memory footprint and integer arithmetic.

## Conclusion

This study demonstrates the large redundancy of the operation of multiplication during the inference of neural networks in popular RL tasks. Minimizing the number of multiplications will prove critical in the areas where computational and energy efficiency are key, for example Edge AI^29^, real-time control^30^, robotics^31–33^, and many others. While suitable equipment to take full advantage of these benefits is currently not available to a consumer, the computational chips capable of evaluating the network inference in the proposed way are currently being researched and developed as a part of neuromorphic computing paradigm. Our study highlights the importance of significant multiplications inference for a plethora of such neuromorphic computing applications.

### Supplementary Information

Supplementary Information 1.

Supplementary Information 2.

Supplementary Tables.

## Data Availability

The data generated or analysed during this study are included in this article and its supplementary information files. Supplementary Tables summarizes the numerical results in all game environments. Supplementary Information 2 contains a model and its neural network weights for the Enduro game (the other weights are available upon request). Supplementary Information 1 contains the source code for the sparse model inference.
