# Supplementary material for: Deep reinforcement learning with significant multiplications inference
Source: Sci Rep. 2023 Nov 27;13:20865. doi: 10.1038/s41598-023-47245-y (PMC10682459; doi:10.1038/s41598-023-47245-y)
Supplement: Supplementary file 3 — Supplementary Tables. [file 41598_2023_47245_MOESM3_ESM.pdf]

Supplementary material for
Ivanov *et. al*, "Deep RL with Significant Multiplications Inference"

Iteration	Default	0	1	2	3	4	5	6	7
Conv2d-1	3276800	117964	101580	86016	72089	59801	52428	44236	37683
Conv2d-2	2654208	199729	187121	120102	89579	68345	59719	37822	29859
Conv2d-3	1806144	405027	332330	174744	118753	114690	116496	67730	50120
Dense-1	1605632	478478	455196	264126	188260	292225	415457	283795	262119
Dense-2	2048	553	529	259	175	279	431	281	278
Total NonZero	9344832	1201751	1076756	645247	468856	535340	644531	433864	380059
NonZero frac	1.000	0.129	0.115	0.069	0.050	0.057	0.069	0.046	0.041

Table S1. Number of multiplications in Freeway

Iteration	Default	0	1	2	3	4	5	6	7
Conv2d-1	3276800	183500	131072	104038	77004	58163	44236	31948	24576
Conv2d-2	2654208	331112	261439	230916	180486	150626	118112	88915	72990
Conv2d-3	1806144	475467	419025	358068	315172	255569	212673	170229	144943
Dense-1	1605632	134873	157753	193478	203513	237633	305070	303063	364478
Dense-2	2048	175	180	205	204	227	222	226	205
Total NonZero	9344832	1125127	969469	886705	776379	702218	680313	594381	607192
NonZero frac	1.000	0.120	0.104	0.095	0.083	0.075	0.073	0.064	0.065

Table S2. Number of multiplications in Enduro

Iteration	Default	0	1	2	3	4	5	6	7
Conv2d-1	3276800	49971	42598	36044	29491	26214	22118	18841	16384
Conv2d-2	2654208	204374	168542	130719	109486	90243	72990	56401	45785
Conv2d-3	1806144	271824	228928	187387	163004	129590	103853	83082	69536
Dense-1	1605632	152133	152133	159358	167788	185450	187457	200302	222781
Dense-2	2048	394	402	461	452	485	510	477	513
Total NonZero	9344832	678696	592603	513969	470221	431982	386928	359103	354999
NonZero frac	1.000	0.073	0.063	0.055	0.050	0.046	0.041	0.038	0.038

Table S3. Number of multiplications in SpaceInvaders

Iteration	Default	0	1	2	3	4	5	6	7
Conv2d-1	3276800	299827	232652	180224	136806	103219	78643	63897	50790
Conv2d-2	2654208	368271	305233	272719	242196	223617	188448	177168	159252
Conv2d-3	1806144	185581	195515	201836	199127	179711	149458	129139	107465
Dense-1	1605632	37732	47767	77070	99549	134471	171802	220372	238436
Dense-2	2048	488	489	494	470	450	461	447	430
Total NonZero	9344832	891899	781656	732343	678148	641468	588812	591023	556373
NonZero frac	1.000	0.095	0.084	0.078	0.073	0.069	0.063	0.063	0.060

Table S4. Number of multiplications in Krull

Iteration	Default	0	1	2	3	4	5	6	7
Conv2d-1	3276800	22118	18022	17203	15564	11468	12288	9830	8192
Conv2d-2	2654208	38486	41803	39813	30523	33841	25878	26542	15261
Conv2d-3	1806144	64118	72697	62311	48314	54184	37929	39735	22576
Dense-1	1605632	39337	56598	75063	73457	100352	85901	126443	91521
Dense-2	2048	114	184	223	201	369	349	492	415
Total NonZero	9344832	164173	189304	194613	168059	200214	162345	203042	137965
NonZero frac	1.000	0.018	0.020	0.021	0.018	0.021	0.017	0.022	0.015

Table S5. Number of multiplications in Breakout

Iteration	Default	0	1	2	3	4	5	6	7
Conv2d-1	3276800	497254	367820	232652	261324	244121	207257	154828	144998
Conv2d-2	2654208	439271	299925	187785	184467	154607	120766	86925	74981
Conv2d-3	1806144	408188	277243	178356	167519	141782	106562	80373	66827
Dense-1	1605632	374112	335175	273760	307478	291020	270147	260513	259710
Dense-2	2048	252	255	243	270	306	303	285	292
Total NonZero	9344832	1719077	1280418	872796	921058	831836	705035	582924	546808
NonZero frac	1.000	0.184	0.137	0.093	0.099	0.089	0.075	0.062	0.059

Table S6. Number of multiplications in Robotank
